# Initial Assessment of VECTRA Three-Dimensional Imaging to Accurately Simulate Breast Volume Changes in Transfeminine Patients: A Mannequin Study

**DOI:** 10.1093/asjof/ojad015

**Published:** 2023-02-15

**Authors:** Ximena Lopez, Jasmine Panton, Purushottam Nagarkar, Stephanie Preston, Jessica Abramowitz, Bardia Amirlak

## Abstract

**Background:**

Methods that aim to accurately measure and predict breast development can be utilized in gender-affirming treatment planning, patient education, and research.

**Objectives:**

The authors sought to evaluate whether three-dimensional (3D) stereophotogrammetry accurately measures transfeminine breast volume changes on a masculine frame when simulating anticipated changes in soft tissue after gender-affirming surgical therapy. Then, we describe the innovative application of this imaging modality in a transgender patient to illustrate the potential role of 3D imaging in gender-affirming surgical care.

**Methods:**

A 3D VECTRA scanner (Canfield, Fairfield, NJ) was used to measure anthropometric breast measurements. Postoperative changes in breast volume were simulated on a cardiopulmonary resuscitation mannequin using 450 cc MENTOR breast implants (Mentor Worldwide LLC, Irvine, CA). To demonstrate the ability of the VECTRA to accurately simulate transfeminizing augmentation in practice, we describe its use in a 30-year-old transgender female with a 2-year history of gender-affirming hormone therapy, presenting for gender-affirming surgical care.

**Results:**

In the mannequin, mean breast volumes were 382 cc on the right (range 375-388 cc), and 360 cc on the left (range 351-366 cc). The average calculated difference in volume between the 2 sides was 22 cc (range 17-31 cc). There were no instances where the left side was calculated to be larger than the right or where the calculated size was smaller than the actual implant size.

**Conclusions:**

The VECTRA 3D camera is a reliable and reproducible tool for preoperative assessment, surgical planning, and simulating breast volume changes after gender-affirming surgery.

**Level of Evidence: 5:**

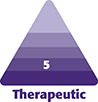

Gender-affirming hormone and surgical therapy have variable effects on breast development. Methods that aim to accurately measure breast development can be utilized in treatment planning, patient education, and research to assess the effect of gender-affirming hormone or surgical threapies on breast changes. In addition, anthropometric measurements are fundamental to the preoperative assessment of patients undergoing aesthetic or gender affirmation breast surgery.

In the past, aesthetic breast surgery results (eg, augmentation, reduction, or lift) have been analyzed by quantifying physician opinion. Plastic surgeons also use patient satisfaction surveys and questionnaires to quantify patient opinion, which tend to be subjective.^1^ Medical photography is a mainstay of two-dimensional (2D) imaging in plastic surgery and provides clinical data points both before and after surgery in addition to being relatively inexpensive.^2^ However, 2D imaging does not offer important topographic measurements such as breast volume, which are being viewed as more essential to surgical planning and aesthetic prediction. Thus, recent emphasis has shifted to three-dimensional (3D), as this technology provides topographic measurement, and can trace subtle changes in breast volume, size, and shape, throughout the course of breast development.^3^

Computed tomography (CT), 3D ultrasonography, moiré topography, laser scanning, and stereophotogrammetry (combining photographs from different angles) are just a few examples of 3D-imaging modalities. The main advantage of 3D scanners using cameras over lasers and CT scans lies in their clinical safety and higher speed of obtaining data.^2^ The demand for virtual surgical planning (VSP) is increasing among surgeons and patients alike. Utilizing either cross-sectional or 3D surface imaging (3D-SI) to better visualize the patient's preoperative state and plan the desired postoperative result has become the standard of care in plastic surgery, particularly with breast augmentation procedures. VSP utilizing multiple fixed cameras range in cost and sophistication from the simple use of 3D images as a discussion and planning tool to the development of 3D-printed models.^1,3,4^

Three-dimensional SI in particular has been described in both aesthetic and reconstructive breast surgery, improving patient satisfaction across a variety of domains.^4^ Patients undergoing gender-affirming chest surgery (masculinizing mastectomy or breast augmentation) would likely experience the same and additional benefits of virtual imaging. Indeed, this tool also has recently been used to trend changes in anthropometric breast measurements during 3 years of gender-affirming hormone treatment in transgender women.^5^

In this preliminary report, we sought to evaluate whether 3D stereophotogrammetry accurately measures transfeminine breast volume changes on a masculine frame when simulating anticipated changes in soft tissue after gender-affirming chest surgery. Then, we describe the innovative application of this imaging modality in a transgender patient to illustrate the potential role of 3D imaging on the gender-affirming surgical care.

## METHODS

### Validation of VECTRA Scanner to Measure Breast Volume

**Figure 1.**
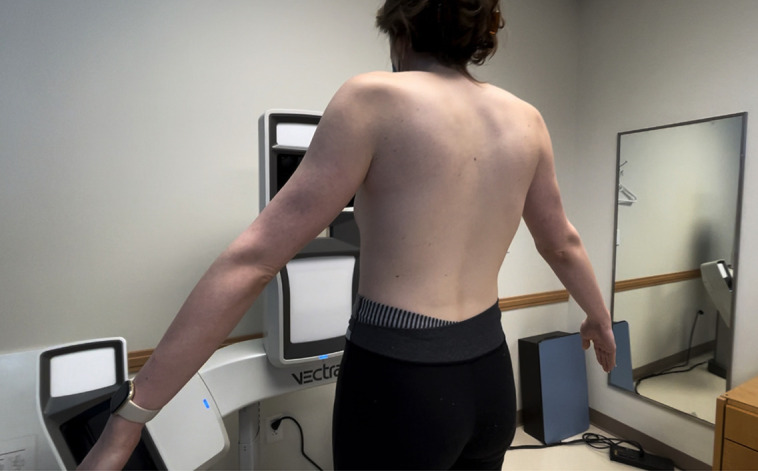
VECTRA 3-dimensional (3D) system (Canfield, Fairfield, NJ) is being used to capture breast images on a 24-year-old transgender female patient.

A 3D VECTRA scanner (Canfield, Fairfield, NJ) was used to measure breast volume (Figure 1). A 24″ × 14″(H × D) Prestan Professional Adult Series 2000 cardiopulmonary resuscitation (CPR) Training Manikin heretofore referred to as “CPR Mannequin” (Prestan, Mayfield Village, OH) was fitted with a form-fitting black shirt to prevent reflection or glare artifacts. MENTOR (Mentor Worldwide, LLC, Irvine, CA) smooth round high profile silicone gel implants were placed bilaterally below the shirt centered on the breast. A black C-cup bra was placed to maintain the position of the implants on the chest wall with the mannequin in vertical position. This allowed precise control of the actual volume differences between the 2 breasts, with the underlying assumption that the mannequin was manufactured with slight asymmetry favoring the left chest.

**Figure 2.**
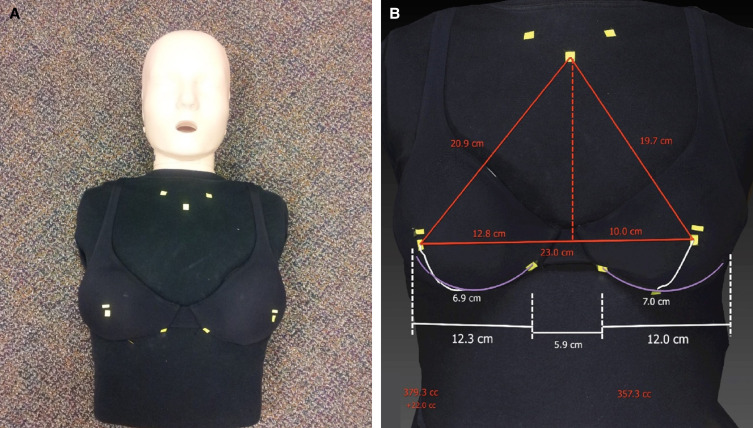
(A) A cardiopulmonary resuscitation (CPR) mannequin with landmarks used by the VECTRA software (Canfield, Fairfield, NJ) with MENTOR implants (Mentor Worldwide LLC, Irvine, CA). (B) Mannequin image captured with VECTRA showing volume, base width, and nipple to inframammary fold distances.

Squares of yellow tape measuring 1 × 1 cm were used to mark the anthropometric landmarks utilized by the VECTRA software. These included the sternal notch, mid-clavicle, nipple, superior areolar border, and the most lateral and medial boundaries of the inframmary fold. On the right, a 375 cc implant was placed (base width 12.0 cm). On the left, a 350 cc implant (base width 11.7 cm) was placed (Figure 2A).

Photos were taken with the mannequin at 3 distances from the VECTRA cameras to simulate potential variations in photographic technique. These were (1) the recommended position with the mannequin's chin at the border of the frame, (2) 30 cm closer to, and (3) 30 cm further from the camera. Each photo was retaken 4 times, and the mannequin was removed from its position and replaced each time, again to simulate potential variability in patient positioning. Measurements were repeated between repositioning attempts to ensure that the mannequin's clothing and implants remained consistent. A total of 12 images were captured in this fashion. The VECTRA calculated the volume, base width, and nipple-IMF distances bilaterally using each image.

### Use of VECTRA-based Virtual Surgical Planning in Transfeminine Patient

To demonstrate the ability of the VECTRA to accurately simulate transfeminizing augmentation, we describe its use in a 24-year-old transgender female with a 6-year history of gender-affirming hormone therapy prior to surgery (Video). She presented for evaluation of breast augmentation surgery to treat symptoms of chest and gender dysphoria. Preoperative simulation VECTRA photos were obtained with a 350 cc moderate plus MENTOR breast implant. Written consent was provided, by which the patients agreed to the use and analysis of their data.

## RESULTS

In the mannequin, the average calculated breast volumes were 382 cc on the right (range 375-388 cc) and 360 cc on the left (range 351-366 cc). The average calculated difference in volume between the 2 sides was 22 cc (range 17-31 cc), which represented an average absolute error of 4 cc (range 0.6-8 cc; Figure 2B). There were no instances where the left side was calculated to be larger than the right. There were no instances where the calculated size was smaller than the actual implant size.

**Figure 3.**
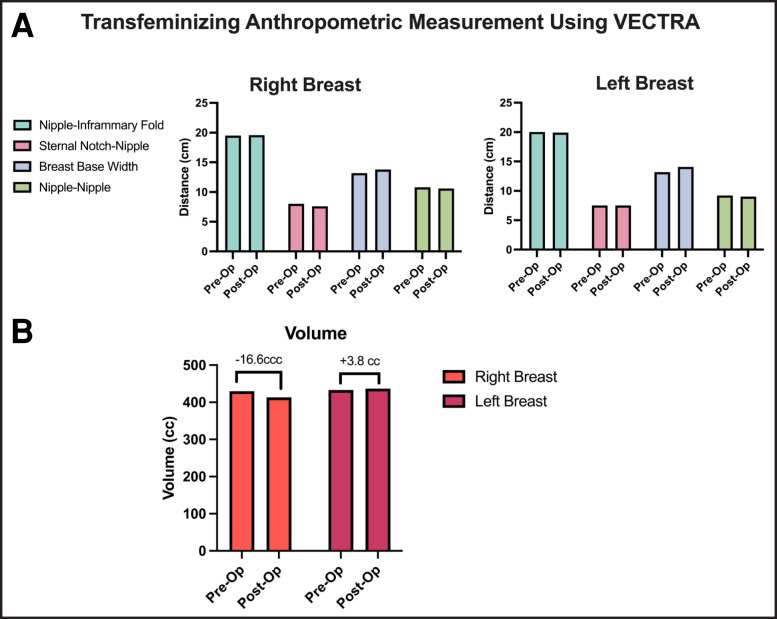
(A) Simulated preoperative and actual postoperative anthropometric measurements in same trans-female patient using VECTRA (Canfield, Fairfield, NJ). (B) Simulated preoperative and actual postoperative breast volume in same trans-female patient using VECTRA.

**Figure 4.**
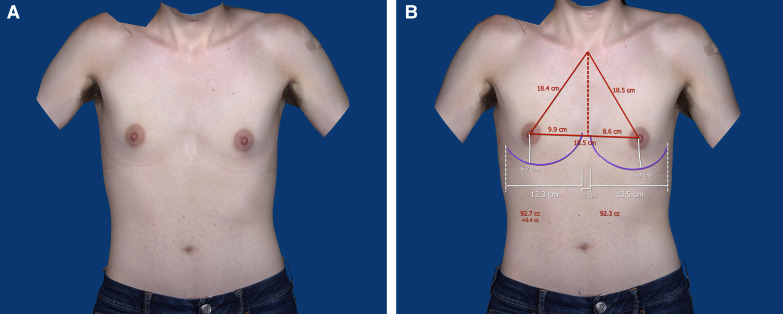
(A) Preoperative photos of a 24-year-old transgender female captured via VECTRA (Canfield, Fairfield, NJ). (B) Preoperative photos of a 24-year-old transgender female with anthropometric measurements captured via VECTRA.

**Figure 5.**
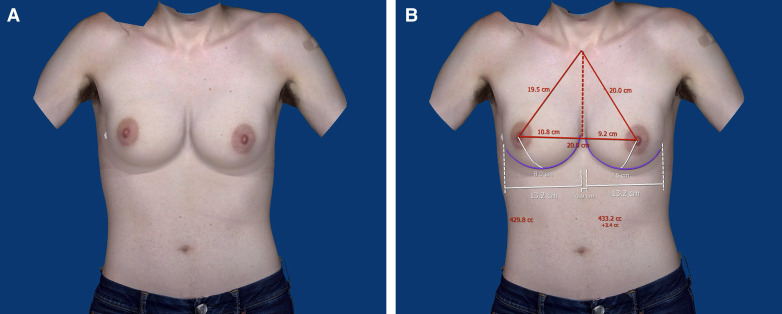
(A) Preoperative VECTRA (Canfield, Fairfield, NJ) photos of the same patient, with transfeminizing augmentation simulated using a 350 cc moderate plus MENTOR implant (Mentor Worldwide LLC, Irvine, CA). (B) Preoperative VECTRA photos and athroprometric measurements of the same patient, with transfeminizing augmentation simulated using a 350 cc moderate plus MENTOR implant.

**Figure 6.**
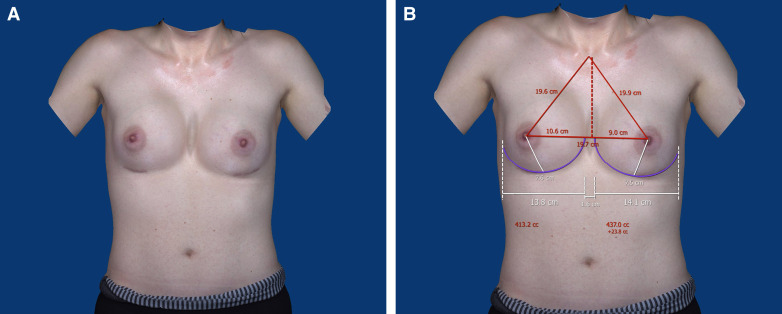
(A) Postoperative VECTRA (Canfield, Fairfield, NJ) photos of the same patient, 8 months following transfeminizing breast augmentation using 350 cc moderate plus MENTOR implant (Mentor Worldwide LLC, Irvine, CA). (B) Postoperative VECTRA photos of the same patient with anthropometric measurements after transfeminizing breast augmentation.

Anthropometric and volumetric measures for a transfeminine patient before and after breast augmentation are summarized in Figure 3A, B, with minimal differences in anatomic landmarks and breast volume capture between preoperative simulation and postoperative measure. Figure 4A, B shows preoperative photos with and without measurements. Figure 5A, B shows the same patient preoperatively with transfeminizing simulation using a 350 cc implant. Figure 6A, B shows postoperative measurements of the same patient following breast augmentation.

## DISCUSSION

There is a clear advantage of 3D over 2D imaging used in numerous surgical fields. 3D-SI technology creates a topographical map by quantifying the interaction of light with a surface. 3D VECTRA scanners are a type of photography-based 3D-SI that involves the use of 3 simultaneous single-lens reflex cameras capable of high-resolution and high-frequency imaging. A multi-camera system is connected to a computer, where the captured data set is saved. Accompanying sculpting software allows for the simulation of a variety of surgical procedures including breast surgeries, rhinoplasty, blepharoplasty, and others. The machine is easily placed in 4 feet by 3 feet space room in office and does not require special lighting (Figure 1).

Despite its clear promise and current application, these technologies are still relatively new and undergoing validation for accuracy in a variety of clinical settings and across a range of clinical indications.^6^ VECTRA has been demonstrated to have good intra- and interobserver variability in breast phantoms, and acceptable but greater variability when it is used to measure human breasts.^6^ It has been used to accurately assess pre-existing asymmetries of the inframammary fold and nipple-areola complex,^7^ as well as those resulting from oncologic breast surgery with immediate reconstruction.^8^

To obtain absolute measurements with 2D photography, one must use a life-sized photograph by recalibrating it to the actual size of the subject and using measurements of known fixed distances. To create the life size photo, a fixed camera to subject interval is also required. This makes obtaining life size results using 2D photographs not only laborious but subjective to the photographer accuracy.^9^ A major advantage of some 3D imaging systems is the ability to perform absolute life size measurements. This ability is useful not only for surgical planning but also for accurate volume measurements during and after gender-affirming hormone therapy.

Our results show that the VECTRA 3D photography and software calculation of volume is quite reliable to identify small differences in volumes, even when accounting for potential variations in photography technique. The calculated volumes here were consistently greater than the actual implant volume, which is not surprising since there was, in fact, additional volume present: The t-shirt and bra used to secure the implants. The difference between the calculated and actual volumes of the implants was ∼5 to 10 cc, which is easily attributable to the volume of these fabrics. The average absolute error in calculated volume difference between the 2 sides was small at 4 cc. As such, the calculated volumes from VECTRA photography can be relied upon to guide surgical and medical decision-making.

VECTRA volumetric analysis has been shown to linearly correlate with that of MRI as well as surgeon estimation of breast volume.^10^ It has also been applied to gauge the changes in breast reconstruction or augmentation mammoplasty results over time, including the development of pseudoptosis and volume loss.^11,12^ Lin et al even observed a correlation between implant volume and N-IMF (nipple to inframammary fold) distance: with each additional 100 mL, the N-IMF distance increased 1 cm.^11^ In a series of 20 patients undergoing augmentation mammoplasty, comparison of VECTRA preoperative simulation to actual postoperative images demonstrated 90.8% volumetric accuracy and an absolute 4 mm root-mean-square difference in surface topography.^13^ Another study also found no significant difference between breast volume in one vs 2-stage implant-based reconstruction.^14^

Importantly, several studies pointed to the potential pitfalls in the use of this technology including the inconsistent definition of the region or interest and developed their own protocols for defining the breast border within the VECTRA software. A persistent challenge is the accurate assessment of the posterior or deep border of the breast, or the chest wall contour. There is no current gold-standard protocol for the volumetric measurement of breast tissue, leading to a lack of generalizability. Other drawbacks include lower accuracy of volumetric measurements in ptotic and larger breasts.^12,13^

While no study has definitively shown that VSP improves outcomes, patients do consistently voice improved confidence in their preoperative counseling and surgeon with the use of VSP.^15,16^ VSP could provide a variety of benefits to transgender patients in particular. For transfeminine patients, VECTRA could aid in quantifying breast hypertrophy over time after initiating estrogen therapy and determining the point at which breast hypertrophy plateaus. Clarification of this clinical endpoint could assist patients in determining whether to pursue breast augmentation or inform timing of the procedure. The relationship between implant volume and breast projection might also differ in transgender compared with cis-gender women due to differences in the breast footprint at baseline. The VECTRA simulation tool allows the patients to see the 3D Holographic results of various sizes and shapes of breast implants. This ability helps to determine the desired implant size based on the breast width, skin envelope, and patient's desire. The software can calculate the existing volume difference between the 2 breasts (Figures 2, 3), as well as points of asymmetry which can be used as an educational tool for the patient. For transmasculine patients, VECTRA could be particularly helpful in visualizing outcomes of periareolar vs double-incision mastectomy techniques such as chest volume and the postoperative nipple-areolar complex position.

There is ample evidence pointing toward the role of gender-affirming hormone therapy in reducing body dissatisfaction.^17^ Two studies have examined the effect of hormone therapy on breast development in adult transwomen, with only centimeter measurement with a tape, bra cup sizes, and Tanner stages used.^18,19^ These methods are highly subjective and subject to intra- and interinterpreter variability. There is a paucity of evidence examining breast development based on change in volume following gender-affirming hormone therapy with estrogen or testosterone in the adult and adolescent transgender population. The ability to measure shape and volume can be used to assess breast development in this nonsurgical setting, as well as breast changes associated with chronic chest binding in transmasculine patients.

In addition, there have been limited studies on the effect of illusory body-sex changes with VSP on transgender care.^20^ As 3D imaging offers patients a realistic portrayal of how they might look after gender-affirming surgical change, these modalities have the potential to affirm gender identity preoperatively and further guide decision-making. For example, mental health response to the hormonal and surgical treatments could potentially be predicted with such simulation tools before the initiation of treatment. We speculate that 3D simulations, virtual reality, and holographic imaging technology could be used in the near future by gender mental health therapists for their initial assessment of the degree and nature of body dysphoria.

## CONCLUSIONS

In the transfeminine patient, the accuracy of anthropometric breast measurements remains a challenge. We propose that the VECTRA offers the ability to accurately simulate postoperative transfeminine augmentation and anticipate feminizing changes in patients seeking gender-affirming medical and surgical care. The VECTRA is an innovative tool which can accurately measure breast topography and volume. When used within acceptable parameters, it is a reliable and reproducible method for preoperative assessment, preoperative planning, simulation of postoperative results, as well as in the measurement of breast volume changes with gender-affirming hormone therapy.

## Supplementary Material

ojad015_Supplementary_Data
